# Assessment of Awareness and Practices Regarding Breast Cancer Among Women of Reproductive Age: A Cross-Sectional Study

**DOI:** 10.7759/cureus.77612

**Published:** 2025-01-18

**Authors:** Santosh D Patil, Muttappa R Gudadinni, Mallikarjun Yadavannavar, Navarathna Murthi

**Affiliations:** 1 Community Medicine, Shri B.M. Patil Medical College, Hospital and Research Center, Vijayapura, IND

**Keywords:** best practice, breast cancer research, reproductive age-group, self-awareness, self-breast examination

## Abstract

Background: Cancer is a major global health challenge, with breast cancer being the most common and serious health concern for women. It is widely recognized as the most frequently diagnosed cancer and a significant cause of mortality among women.

Objectives: The objective of this study is to evaluate awareness and practices related to breast cancer. As well as, it also aimed to identify gaps in knowledge and practices to guide the development of effective education and training programs for improving awareness, early detection and prevention of breast cancer among women of reproductive age.

Materials and methods: A cross-sectional study was conducted in an urban area over six months in 2024. Women aged 18-49 years were included, with a total sample size of 100. Data were collected using a structured, self-administered questionnaire focusing on socio-demographic factors, awareness of breast cancer risk factors, preventive practices and screening procedures.

Results: The study revealed that most participants (46, 46%) were aged 18-30 years, and 51 (51%) were married. A majority (56, 56%) belonged to the Hindu religion. The findings indicated that 55 (55%) of participants were aware of breast self-examination (BSE), 42 (42%) knew the recommended frequency for performing BSE, and 31 (31%) were aware of the appropriate age to start BSE. Only 36 (36%) of participants performed BSE regularly on a monthly basis, and 43 (43%) reported observing differences between their breasts during BSE.

Conclusion: The identified gaps in awareness and the limited practice of BSE among participants highlight the importance of targeted educational efforts to enhance breast cancer awareness and promote primary prevention strategies.

## Introduction

Globally, cancer accounts for 12% of all deaths [1]. Breast cancer is the most commonly diagnosed cancer and the leading cause of cancer-related death among women worldwide [2]. It is a pervasive and life-threatening disease that disproportionately affects women, posing significant risks to their health and well-being [3]. In the United States, breast cancer is the most commonly diagnosed cancer and ranks as the second leading cause of cancer-related deaths among women [4]. Similarly, in India, breast cancer is the most prevalent cancer among women, contributing to around 14% of all female cancer cases. In 2020, India recorded an estimated 178,361 new breast cancer cases and 90,408 deaths associated with the disease [5].

Breast cancer develops from the abnormal growth of cells in breast tissue, leading to malignant tumors. Characterized by uncontrolled cell growth, these malignant tumors can spread to nearby tissues and distant parts of the body [6]. The incidence of breast cancer is increasing in developing countries, driven by lifestyle changes and longer life spans. However, limited healthcare resources and weak health systems often result in delayed diagnoses. Raising awareness about early detection, including breast self-examination (BSE), is crucial to addressing this disparity [7].

The American Cancer Society and other health organizations recommend various breast cancer screening methods, including monthly BSE, clinical breast examination (CBE) by a healthcare professional, and mammography, which involves annual or biennial X-ray imaging [8]. In low-income countries, BSE is a vital screening tool due to limited access to routine health check-ups and the high cost and intimidating nature of mammography services. Currently, 85% of patients in such settings seek specialized care only after tumors have grown beyond 5 cm. In contrast, BSE can help detect tumors as small as 1 cm, enabling earlier intervention. Effective Information, Education, and Communication (IEC) strategies are essential for recognizing abnormal breast conditions and promoting early detection through BSE [9]. By emphasizing BSE and IEC, we can improve breast cancer outcomes, reduce late-stage diagnoses, and enhance health equity in resource-constrained settings. BSE fosters self-awareness, facilitating the early detection of breast tumors when treatment options are most effective [10].

Recent trends indicate that breast cancer has become the most prevalent cancer among Indian women, surpassing cervical cancer, particularly in urban areas. The prevalence rate of breast cancer in India is approximately 130.19 per 100,000 women [11]. This trend is attributed to lifestyle changes and increased life expectancy [12]. Indian women often present with breast cancer at advanced stages, significantly lowering survival rates. In contrast, early-stage diagnosis improves survival chances, prognosis, and treatment outcomes [13].

Despite ongoing cancer awareness campaigns, India lacks a dedicated national program specifically targeting breast cancer. Awareness among women remains suboptimal despite strong interest in the subject. Implementing effective breast cancer education and awareness programs tailored to the Indian community is critical for promoting early detection, timely treatment, and improved survival rates. This study undertakes a comprehensive review to evaluate the level of awareness and practices regarding breast cancer and its screening modalities among the Indian population.

Objectives

The objective of this study is to determine the level of awareness and practices regarding breast cancer among women of reproductive age and to identify areas for improvement in order to develop effective education and training programs aimed at enhancing breast cancer awareness, early detection, and prevention.

## Materials and methods

Study design and period

A cross-sectional study was conducted from January to June 2024.

Study area

The study was carried out in the urban field practice area of Chandabowdi, Vijayapura, India.

Study participants and sample size

The study focused on women of reproductive age (18-49 years) residing in the designated urban area. The sample size was determined using the formula $N = \frac{4pq}{l^2}$​ [14], where p=0.56 (awareness level from a pilot study), q=1-p, and l=10% (absolute error). The calculated sample size was 98.56, rounded up to 100 participants. Participants were selected using purposive sampling to ensure representation within the reproductive age group.

Data collection

Data were gathered using a structured, pre-tested, and self-administered questionnaire (in the local Kannada language) validated through a pilot study to ensure accuracy and reliability. Prior to data collection, participants received a detailed explanation of the study's purpose, objectives, procedures, potential risks, and benefits. Informed verbal consent was obtained from each participant, and institutional ethical clearance was secured before the study's initiation (no: BLDE(DU)/IEC/1100-D/2023-24).

A house-to-house survey was conducted, employing systematic random sampling to select participants. The questionnaire was designed to capture data across five key domains: socio-demographic characteristics, awareness of risk factors, breast cancer-related practices, preventive measures, and screening procedures.

Inclusion and exclusion criteria

The study included women aged 18-49 years residing in the urban area who were willing to participate. The exclusion criteria for the study were as follows: migratory populations and individuals with chronic psychosocial conditions.

Statistical analysis

The data were presented using numbers and percentages and results were presented in tabular form for clarity and ease of interpretation. These were done using the Statistical Package for the Social Sciences (IBM SPSS Statistics for Windows, IBM Corp., Version 26.0, Armonk, NY)

## Results

From Table 1, we observed that a maximum of 46 (46%) of the study group were aged 18-30 years, while 16 (16%) of subjects were aged over 41 years. Fifty-one (51%) of the study group were married, and nine (9%) were separated. The religious affiliation showed a significant concentration of Hindu participants, that is, 56 (56%). A maximum of 37 (37%) educational status of subjects had up-to-primary-level education, 25 (25%) had higher secondary education, and the lowest (four, 4%) had a degree. Approximately one-third of the study group, i.e., 49 (49%) belonged to class IV, 39 (39%) belonged to class III, and 12 (12%) to class V, according to Prasad's classification. Almost half, 39 (39%), of the subjects were housewives, 27 (27%) were daily wage earners, and the least, four (4%), were government employees.

**Table 1:** Distribution of respondents related to socio-demographic variables

Variables		N=100	
		N	%
Age	18-30	46	46
	31-40	38	38
	41 and above	16	16
Religion	Hindu	56	56
	Muslim	44	44
Marital status	Married	51	51
	Unmarried	28	28
	Widow	12	12
	Separated	9	9
Occupation	Housewife	39	39
	Daily wagers	27	27
	Salaried govt.	4	4
	Salaried private	20	20
	Business	10	10
Educational status	Illiterate	18	18
	Primary	37	37
	Higher Secondary	25	25
	Pre-university course (PUC)	16	16
	Degree or Postgraduate	4	4
Socioeconomic status	Class V	12	12
	Class IV	49	49
	Class III	39	39

From Table 2, it is revealed that 55 (55%) of the participants were aware of BSE, 42 (42%) of the study participants knew the recommended frequency for performing BSE, and 31 (31%) were the appropriate age to start BSE. A total of 38 (38%) of the study participants identified common breast cancer causal factors, and 32 (32%) were aware of symptoms of breast cancer. Forty-three (43%) of the study participants identified groups for developing a higher risk of breast cancer and 12 (12%) mentioned risk factors among family members. Thirty-eight (38%) of study participants knew the necessary action to take upon finding a breast lump, and 29 (29%) were aware of recommended practices for maintaining breast health.

**Table 2:** Breast cancer awareness among participants

Variables	Correct answered	Percentage (%)
Are you aware of or ever heard of Breast Self-Examination (BSE)?	55	55
How often should women typically perform self-breast examination?	42	42
Appropriate age to perform BSE	31	31
Common risk factors for breast cancer	38	38
Common symptoms of breast cancers	32	32
Which method is used for early detection?	24	24
Has anyone in your family had breast cancer?	12	12
Which of the groups is at a higher risk for developing breast cancer?	43	43
If you find lump in your breast what is the recommended action?	38	38
What is a recommended practice for maintaining breast health?	29	29

From Table 3, it was slightly disappointing to observe that 35 (35%) respondents knew how to properly perform BSE and 40 (40%) of participants performed regular BSE, with varying frequencies. Thirty-six (36%) of participants execute BSE at the same time each month and 43 (43%) of participants looked for differences between their breasts during BSE. Only 24 (24%) of participants correctly placed their hands on their hips and pulled their elbows forward during BSE. Thirty (30%) participants checked for abnormalities of the nipple during BSE.

**Table 3:** Participants practice about the breast cancer

Variables	Correct answered	Percentage (%)
Proper techniques for conducting a Breast Self-Examination (BSE)	35	35
Do you regularly perform BSE and How frequently do you conduct BSE?	40	40
Do you perform BSE same time each time?	36	36
Do you check for differences between your breasts?	43	43
Do you place hands on your hips and then pull your elbows forward?	24	24
Do you look for any abnormalities of nipple?	30	30

**Figure 1:**
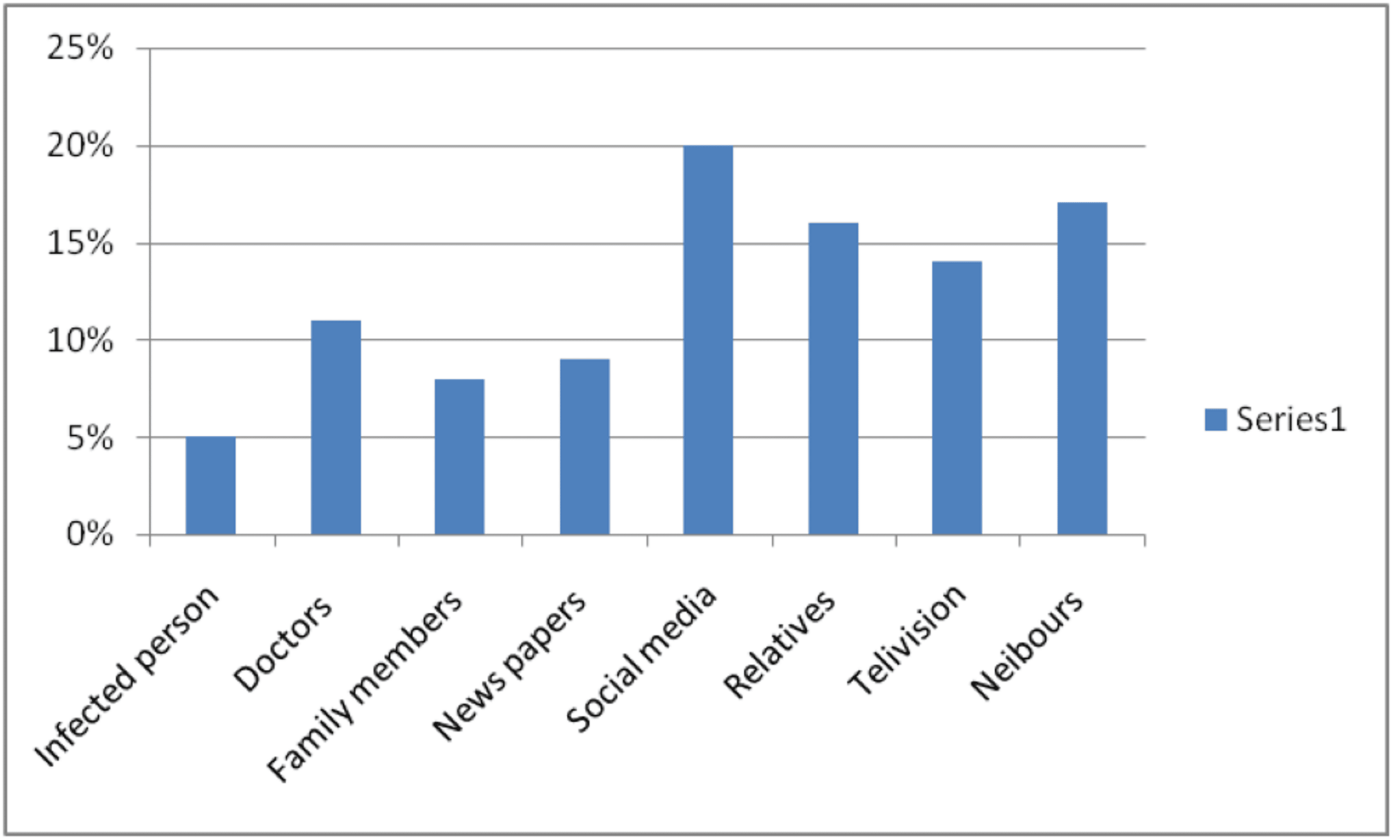
Participant-reported sources of breast cancer information

From Figure 1, we see the Sources of Breast Cancer Information Reported by the study group where almost 20 (20%) and 17 (17%) identified social media and neighbors, respectively, as the higher number of information sources. In addition, relatives 16 (16%) and 14 (14%) television were also leading sources of information. On the other hand, 11 (11%) had known from doctors and five (5%) from infected persons. Additionally, eight (8%) participants learned about breast cancer from family members and nine (9%) from newspapers.

## Discussion

Bridging the Gap

Early recognition of breast cancer is crucial to reduce mortality rates and improve treatment outcomes in India. Despite the importance of early detection, India lacks a national breast cancer screening program, making it vital to raise awareness about BSE, especially among vulnerable populations. Promoting the regular practice of BSE can empower women to detect potential abnormalities early, facilitating timely medical intervention. This study was undertaken to assess the awareness and practices of BSE among reproductive-age females in India to identify key areas for health education and to bridge existing knowledge gaps.

The majority of the study participants were between the ages of 18 and 30 years 46 (46%), with 16 (16%) being over the age of 40. Most participants were married, and 37 (37%) identified as Hindu. Educational levels varied, with 37 (37%) having received education up to the primary level. Socioeconomic status, based on Prasad's classification, showed that 49 (49%) of participants belonged to class VI, highlighting the economic challenges faced by many respondents. These findings align with similar research, such as the study by Choudhary et al. [15], where the age group of 20-30 years accounted for 42 (42%) of participants, and 71 (71%) were married, further confirming trends in demographic characteristics.

The findings reveal significant gaps in breast cancer awareness and knowledge about BSE practices. While 55 (55%) of participants were aware of BSE, only 42 (42%) knew the recommended frequency for performing BSE, and just 31 (31%) knew the appropriate age to start performing it. These findings are consistent with studies by Somdatta and Baridalyne [16] and Rehman et al. [17], which also emphasized the need for increased awareness and education. Only 38 (38%) of respondents were able to identify common risk factors for breast cancer, and 32 (32%) were aware of its symptoms. Additionally, 43 (43%) recognized the higher-risk groups for breast cancer, and 38 (38%) knew what actions to take if they found a breast lump. These findings echo those of Madhukumar et al. [18], which highlighted the discrepancy between general awareness of breast cancer and detailed knowledge about its risk factors and symptoms.

Only 40 (40%) of participants performed regular BSE, with 35 (35%) knowing how to perform it correctly. These findings are consistent with studies conducted by Kommula et al. [19] and Ahmed et al. [20], which also found that a significant proportion of participants were not regularly practicing BSE or did not know how to perform it properly. For instance, a study by Dechasa et al. [21] revealed that while 47.2% of participants had practiced BSE at some point, only 23.36% performed it regularly and correctly. These findings highlight the need for targeted interventions to teach proper BSE techniques and to encourage regular self-examination. Social media and relatives were the primary sources of information about breast cancer for most participants. This underscores the role of social media and community networks in spreading awareness about health issues, including breast cancer. A study by Prusty et al. [22] in India also highlighted the impact of television in enhancing awareness among participants. These findings suggest that leveraging digital platforms and community connections can significantly increase awareness and foster early detection practices.

This study is crucial in understanding the awareness levels and practices related to breast cancer and BSE among reproductive-age females in India. The findings point to significant gaps in both knowledge and practice, highlighting the need for comprehensive education programs aimed at improving awareness of breast cancer risk factors, symptoms, and the correct techniques for performing BSE. By addressing these gaps, the study emphasizes the importance of early detection in reducing breast cancer mortality rates and enhancing overall health outcomes. This research calls for policy interventions to incorporate breast cancer education into health programs, particularly targeting vulnerable populations with limited access to information and healthcare services. By increasing awareness through social media, community outreach, and educational resources, women can be empowered to take proactive steps in their health, ultimately improving early detection and reducing the burden of breast cancer in India.

This study recommends the implementation of community-based awareness programs and health education initiatives that emphasize early detection, prevention, and personal hygiene practices. These measures can improve breast health management, support primary prevention, and reduce breast cancer mortality rates by ensuring early detection and the prompt initiation of treatment. Recommendations for policymakers include establishing national breast cancer awareness campaigns, integrating breast cancer education into school curricula, implementing national breast cancer screening programs, promoting community-based health education programs, and providing financial incentives for breast cancer education initiatives. Actionable recommendations for leveraging social media for breast cancer awareness and education involve developing targeted educational campaigns, collaborating with influencers, sharing testimonies and real-life stories, and promoting self-examination awareness to reach a broader audience effectively.

Our study was limited to the urban population, which inherently benefits from greater access to social media and digital platforms for health awareness. This focus excludes rural populations, where social media influence is minimal, and traditional barriers to accessing breast cancer awareness and education are more pronounced.

## Conclusions

The awareness gap and suboptimal BSE practices among participants underscore the need for targeted educational initiatives to enhance breast cancer awareness and facilitate primary prevention. The findings indicate a clear requirement for educational interventions to improve knowledge of breast cancer risk factors, symptoms, and management, thereby supporting rapid diagnosis and prevention. Enhancing breast cancer awareness through community-level initiatives effectively improves understanding of symptoms, diagnostic methods, and preventive measures. Health education plays a crucial role in promoting personal hygiene and self-examination practices among young women. This study highlights the importance of targeted educational interventions to raise breast cancer awareness and encourage BSE practices.

The study recommends the implementation of community-based awareness programs and health education initiatives that emphasize early detection, prevention, and personal hygiene practices. These measures can improve breast health management, support primary prevention, and reduce breast cancer mortality rates by ensuring early detection and the prompt initiation of treatment.

## References

[1] Park K (2015). Park’s Textbook of Preventive and Social Medicine. 23rd ed.

[2] Bray F, Ferlay J, Soerjomataram I, Siegel RL, Torre LA, Jemal A (2018). Global cancer statistics 2018: GLOBOCAN estimates of incidence and mortality worldwide for 36 cancers in 185 countries. CA Cancer J Clin.

[3] Suh MA, Atashili J, Fuh EA, Eta VA (2012). Breast self-examination and breast cancer awareness in women in developing countries: a survey of women in Buea, Cameroon. BMC Res Notes.

[4] DeSantis C, Siegel R, Jemal A (2015). Breast Cancer Facts and Figures 2015-2016.

[5] Mehrotra R, Yadav K (2022). Breast cancer in India: present scenario and the challenges ahead. World J Clin Oncol.

[6] (2006). The Cancer, National Encyclopedia Bangladesh. http://www.banglapedia.org/httpdocs/HT/C_0033.HTM.

[7] (2024). Breast cancer risk factors. Breast cancer: prevention and control. http://www.who.int/cancer/detection/breast_cancer/en/index2.html.

[8] Hallal JC (1982). The relationship of health beliefs, health locus of control, and self concept to the practice of breast self-examination in adult women. Nurs Res.

[9] Mazzini CB (2016). Knowledge and practice of the breast self-exam among students from a public university in Lima. Arch Cancer Res.

[10] Singh YP, Sayami P (2009). Management of breast cancer in Nepal. JNMA J Nepal Med Assoc.

[11] International Agency for Research on Cancer (2021). GLOBOCAN 2020: Cancer Incidence, Mortality, and Prevalence Worldwide. GLOBOCAN.

[12] Ramakant P, Singh KR, Jaiswal S (2018). A survey on breast cancer awareness among medical, paramedical, and general population in North India using self-designed questionnaire: a prospective study. Indian J Surg Oncol.

[13] Lannin DR, Wang S (2017). Are small breast cancers good because they are small or small because they are good?. N Engl J Med.

[14] Kumar M, Srivastava DJ, Jain PK, Kumar S, Dixit AM, Yadav R (2017). A comparative assessment of knowledge and awareness regarding breast cancer among women of reproductive age group in district Etawah. Natl J Community Med.

[15] Choudhary M, Mohanasundari SK, Ara M (2024). Breast self-examination: knowledge, awareness, and practices among females of reproductive age group. Int J Res Med Sci.

[16] Somdatta P, Baridalyne N (2008). Awareness of breast cancer in women of an urban resettlement colony. Indian J Cancer.

[17] Rehman MA, Tahir E, Ghulam Hussain H (2024). Awareness regarding breast cancer amongst women in Pakistan: a systematic review and meta-analysis. PLoS One.

[18] Madhukumar S, Thambiran UR, Basavaraju B, Bedadala MR (2017). A study on awareness about breast carcinoma and practice of breast self-examination among basic sciences' college students, Bengaluru. J Family Med Prim Care.

[19] Kommula ALSD, Borra S, Kommula VM (2014). Awareness and practice of breast self-examination among women in South India. Int J Curr Microbiol App Sci.

[20] Ahmed WR, Hossny EK, Mohammed GT (2024). Risk assessment tool of breast cancer and barriers against breast self-examination among nurses. Nurs Forum.

[21] Dechasa DB, Asfaw H, Abdisa L (2022). Practice of breast self-examination and associated factors among female health professionals working in public hospitals of Harari regional state: Eastern Ethiopia multicenter study. Front Oncol.

[22] Prusty RK, Begum S, Patil A, Naik DD, Pimple S, Mishra G (2020). Knowledge of symptoms and risk factors of breast cancer among women: a community based study in a low socio-economic area of Mumbai, India. BMC Womens Health.

